# Similarity Gait Networks with XAI for Parkinson’s Disease Classification: A Pilot Study

**DOI:** 10.3390/bioengineering13020151

**Published:** 2026-01-28

**Authors:** Maria Giovanna Bianco, Camilla Calomino, Marianna Crasà, Alessia Cristofaro, Giulia Sgrò, Fabiana Novellino, Salvatore Andrea Pullano, Syed Kamrul Islam, Jolanda Buonocore, Aldo Quattrone, Andrea Quattrone, Rita Nisticò

**Affiliations:** 1 Neuroscience Research Center, Department of Medical and Surgical Sciences, Magna Graecia University of Catanzaro, 88100 Catanzaro, Italy; camilla.calomino@unicz.it (C.C.); mariannacrasa@unicz.it (M.C.); alessia.cristofaro@unicz.it (A.C.); giulia.sgro@unicz.it (G.S.); f.novellino@unicz.it (F.N.); jolanda.buonocore@unicz.it (J.B.); quattrone@unicz.it (A.Q.); an.quattrone@unicz.it (A.Q.); r.nistico@unicz.it (R.N.); 2 Laboratory of Biomedical Applications Technologies and Sensors (BATS), Department of Health Sciences, Magna Graecia University of Catanzaro, 88100 Catanzaro, Italy; pullano@unicz.it; 3 Department of Electrical Engineering and Computer Science, University of Missouri, Columbia, MO 65211, USA; islams@missouri.edu; 4 Institute of Neurology, Department of Medical and Surgical Sciences, Magna Graecia University of Catanzaro, 88100 Catanzaro, Italy

**Keywords:** wearable device, Parkinson’s disease, machine learning, morphological similarity networks, graph analysis

## Abstract

Parkinson’s disease (PD) is characterized by alterations in movement dynamics that are difficult to quantify with conventional clinical assessment. This study proposes an integrated approach combining graph-based kinematic analysis with explainable machine learning to identify digital biomarkers of Parkinsonian motor impairment. Kinematic signals were acquired using Xsens inertial sensors from 51 patients with PD and 53 healthy controls. For each participant, subject-specific kinematic networks were constructed by modeling inter-segment similarities through Jensen–Shannon divergence, from which global and local graph-theoretical metrics were extracted. A machine learning pipeline incorporating voting feature selection, and XGBoost classification was evaluated using a nested cross-validation design. The model achieved robust performance (AUC = 0.87), and explainability analyses using SHAP identified a subset of 13 features capturing alterations in velocity, inter-segment connectivity, and network centrality. PD was characterized by increased positional variability, reduced distal limb velocity, and a redistribution of network centrality towards proximal body segments. These features were associated with clinical severity, confirming their physiological relevance. By integrating graph-theoretical modeling, explainable artificial intelligence, and machine learning methodology, this work provides a method of discovering quantitative biomarkers capturing alterations in motor coordination. These findings highlight the potential of ML and kinematic networks to support objective motor assessment in PD.

## 1. Introduction

Parkinson’s disease (PD) is a progressive neurodegenerative disorder mainly characterized by motor symptoms such as bradykinesia, tremor, and postural instability [1]. Bradykinesia is a progressive reduction of movement speed due to dopamine depletion, and it is a primary diagnostic criterion for Parkinsonism [2]. Despite being the diagnostic gold standard, clinical evaluation by neurologists is subjective and constrained to the clinical environment, limiting its ability to reflect the motor performance of the patients [3].

In this context, wearable sensing technologies have attracted increasing attention as reliable tools for objectively quantifying motor impairments in Parkinson’s disease [4,5,6]. Among them, inertial measurement units (IMUs) enable high-resolution acquisition of kinematic signals—including position, velocity, and acceleration—thus providing valuable insights into motor performance with minimal invasiveness [7]. In PD, asymmetry in the motor manifestations of the upper and lower limbs represents a distinctive feature, while gait velocity is markedly reduced in most patients [8]. Traditionally, gait and motor kinematics have been examined through analyses focusing on individual segments or by condensing complex motion into a limited set of summary parameters [9]. While informative, this approach inevitably overlooks the interdependencies that define human movement as an integrated system. To address this limitation, complex network theory has emerged as a powerful mathematical framework for representing and quantifying relationships among multiple interacting components within a system [10,11]. By modeling movement data as networks—where nodes correspond to body segments or sensors and edges encode their interdependencies—graph analysis enables the investigation of both global network organization and the contribution of individual elements to overall motor coordination [10]. Building on this premise, combining graph-theoretical analysis with wearable sensing technologies offers a promising approach to the objective, data-driven characterization of Parkinson’s disease. Moreover, machine learning techniques have demonstrated strong potential for identifying motor abnormalities and distinguishing pathological movement patterns from healthy ones [12,13,14]. In this context, recent work such as ChiGa-Net has shown that combining statistically refined deep feature extraction with a genetically optimized neural network can improve disease detection and generalizability across datasets [15]. This work aims to integrate graph-based kinematic analysis with explainable machine learning to identify objective digital biomarkers of Parkinsonian motor impairment. In particular, position and velocity signals acquired from Xsens inertial sensors are extracted and analyzed, followed by the application of machine learning algorithms. This study has the potential to pave the way for more objective assessments that automatically characterize and classify movement patterns or disease-related alterations in PD compared with healthy controls.

### Related Works

IMU-based gait analysis is performed to classify Parkinson’s disease using conventional machine learning methods (SVM, random forests, k-NN). However, these approaches treat sensor signals as independent channels, ignoring the spatial and temporal dependencies that naturally exist between body segments. In addition to time-domain representations, frequency-domain features have been widely used in sensor-based machine learning studies to characterize signal periodicity and oscillatory components, including applications in movement and vibration analysis [16,17]. Recent studies have highlighted the relevance of graph-based approaches for modeling human movement, particularly when the goal is to quantify coordination among physiological or biomechanical subsystems during motor tasks [14,18,19]. A seminal contribution in this field is the work of Naro et al., who introduced the concept of muscle networks to investigate the reorganization of neuromuscular connectivity during gait in patients with neuromuscular disorders [20]. Electromyographic activity from multiple lower-limb muscles was used to construct adjacency matrices that express intermuscular coupling. From these matrices, the authors extracted graph theory metrics (including network density, modularity, and clustering coefficient) to quantify how groups of muscles interact as functional units during movement. The study demonstrated that pathological motor behavior is reflected in altered network topology, revealing compensatory patterns and loss of synchronization among muscles.

The “kinectome” formalizes whole body movement as a network in which body points are nodes and inter-point kinematic couplings define edges, enabling the detection of asymmetries and coordination changes in health and Parkinson’s disease (PD). This framework demonstrates that network topology derived from kinematics carries physiologically meaningful information beyond conventional segment-wise metrics. More recently, graph-based frameworks have been extended to data acquired from wearable sensors. Rashnu and Salimi-Badr introduced a hybrid deep learning architecture combining 1D-CNNs, GRUs, and graph neural networks (GNNs), where each pressure or inertial sensor was represented as a node, and edges modeled the physical relationships among sensors positioned on the foot [21]. The GNN component enabled the model to learn inter-sensor relationships and encode the gait cycle as a structured graph rather than a simple time series. This representation substantially improved PD classification accuracy, demonstrating that graph-based modeling captures aspects of gait dynamics that are not recoverable from traditional feature-based analysis. Taken together, these studies show that graph representations—whether derived from electromyographic activity or wearable motion sensors—provide a powerful theoretical framework to characterize movement as an integrated system.

## 2. Materials and Methods

### 2.1. Subjects and Clinical Evaluation

Fifty-one subjects with Parkinson’s disease (PD) and fifty-three healthy controls (HC) were included in this research. PD patients were consecutively recruited from 2022 to 2025 at the Institute of Neurology, University Magna Graecia of Catanzaro, Italy. The diagnosis of PD was established according to the latest international clinical diagnostic guidelines [22]. All PD participants underwent a comprehensive neurological assessment in the practical “off” state (after overnight medication withdrawal), conducted by the same specialist in movement disorders. This evaluation included the Movement Disorder Society-sponsored revision of the Unified Parkinson’s Disease Rating Scale (MDS-UPDRS) [23] and the Hoehn and Yahr (H-Y) staging scale. Additionally, cognitive assessments, 123I-FP-CIT-SPECT imaging, and 3T brain MRI scans were performed. Exclusion criteria for PD patients included clinical signs suggestive of alternative diagnoses, normal striatal uptake on 123I-FP-CIT-SPECT, and MRI findings indicative of neoplasms, PSP, multiple system atrophy, normal pressure hydrocephalus, or extensive subcortical vascular changes, including lacunar infarcts in the basal ganglia. The healthy controls had no history of neurological, psychiatric, or significant medical conditions.

### 2.2. Data Acquisition and Preprocessing

The Xsens MVN inertial motion capture system is used to study the gait patterns of PD and HC. The MVN setup consists of compact inertial sensors, wireless communication, enabling comprehensive full-body motion capture. Each motion tracker (MT) measures 3D angular velocity with gyroscopes, 3D acceleration through accelerometers, and 3D magnetic field strength using magnetometers, while barometric pressure is obtained from a built-in barometer. The fusion of the Xsens algorithms with the MT units ensures precise, drift-free 3D orientation estimation, which is particularly beneficial for tracking the orientation of individual body segments. The MT units are highly suitable for this purpose, as they guarantee accurate time synchronization of sensor readings across multiple wireless modules. This is an essential requirement for reliable joint angle measurements. A LiPo battery supplies power, and data transmission occurs through an optimized 2.4 or 5.0 GHz spread-spectrum wireless link to an access point connected to a PC, or alternatively via Ethernet. The wireless range extends up to 20–50 m, with an operating frequency between 2405 MHz and 2475 MHz and a rated RF output of 2 dBm. The system supports a maximum update rate of 60 Hz [24]. Participants were equipped with a set of 17 wireless MT. The body segment dimensions of each participant were recorded during the baseline assessment session. In the experimental task, participants were instructed to walk forward for 10 consecutive steps, make a right turn, and return to their starting point. The task was repeated once. Subsequently, the same procedure was repeated, this time with a left turn. Kinematic data were collected during a walking task consisting of 10 consecutive steps using a 3D motion capture system. The following segments, pelvis, L5, L3, T12, T8, neck, head, bilateral shoulder, upper/forearm, hand, upper/lower leg, foot, and toe were analyzed, and each trial recorded the Cartesian coordinates (x, y, z) of the relevant body joints. Signals were preprocessed by applying a 4th-order Butterworth low-pass filter (cut-off 10 Hz, sampling rate 100 Hz) with zero-phase filtering (*filtfilt*) to reduce high-frequency noise. For each segment, the Euclidean magnitude was computed:

$$m_{i}(t)=\sqrt{x_{i}^{2}(t)+y_{i}^{2}(t)+z_{i}^{2}(t)}$$

This step provided a scalar representation of joint displacement, minimizing orientation effects and sensor noise. From the filtered kinematic trajectories, position and velocity signals were derived. Subsequently, a single statistical descriptor—the interquartile range (IQR)—was extracted to quantify signal variability and amplitude dispersion: IQR = Q3 − Q1 where Q1 and Q3 are the first and third quartiles of the signal amplitude distribution. This feature captures the central spread of motion while being robust to outliers and noise.

### 2.3. Graph-Theoretical Feature Construction

Following the preprocessing of the kinematic signals, individual movement networks were constructed using a distribution-divergence-based framework inspired by recent studies on probabilistic similarity metrics [25]. In this context, each body segment (or joint) was represented as a node, and the edges quantified the degree of similarity between the temporal dynamics of segment signals. Subject-specific movement networks were derived only from the position magnitudes. Specifically, the connections between nodes were estimated by comparing the probability distributions of the amplitudes of the position and velocity signals across each pair of segments. For each segment i and j, the Jensen–Shannon divergence (JS) was computed as:

$$JS(p_{i},p_{j})=\;\frac{1}{2}[\;KL(p_{i}∥M)+KL(p_{J}∥M)],\;M=\frac{1}{2}(p_{i}+p_{J})$$

where pi(t) and pj(t) are the probability density functions (PDFs) of the signal distributions from segments i and j, respectively, and KL(⋅∥⋅) denotes the Kullback–Leibler divergence. The PDFs were estimated via kernel density estimation (KDE) [26], allowing a smooth representation of signal variability. In this framework, a lower JS divergence indicates a higher similarity between the two distributions, corresponding to stronger functional resemblance in their movement patterns. Consequently, adjacency matrix of each subject was built by converting divergence values into connection strengths, such that:

$$A_{ij}=1-JS(p_{i},p_{j})$$

This yielded a weighted connectivity representation of inter-segment relations, which subsequently underwent adaptive thresholding (0.20–0.60, step = 0.05) to derive binary networks for graph-theoretical analysis. The use of thresholding and binarization is a standard procedure to reduce the influence of spurious connections on network topology [27,28]. Network metrics were computed across the full threshold range and averaged to obtain robust, threshold-independent estimates. From each subject’s graph (NetworkX) we computed local metrics: degree, strength (sum of edge weights), clustering, betweenness, closeness (treating weight as distance), eigenvector centrality, and PageRank, and global metrics: density, average clustering, global efficiency, characteristic path length (on the largest connected component when necessary), modularity (greedy communities), assortativity, transitivity, average strength, and number of connected components. Age and sex were regressed out from all extracted features, including kinematic measures and graph-theoretical metrics, using linear residualization prior to machine learning analysis, to minimize potential confounding effects.

### 2.4. Machine Learning Models

A preprocessing pipeline was designed to ensure data consistency and reproducibility. All extracted features were standardized prior to model training. Columns with more than 80% missing values were excluded, remaining missing entries were imputed using the median, and features with zero variance were removed. Each feature was then standardized to a zero mean and unit variance. To identify the most discriminative and stable subset of features, we implemented a voting feature selection (VFS) strategy. The selector used: ANOVA F-values, L1-regularized logistic regression coefficients, XGBoost feature importances, and MultiSURF. The goal of this approach is to combine univariate, linear multivariate, non-linear, and interaction-aware feature relevance measures, mitigating bias toward any single model assumption. Specifically, VFS aggregates the outcomes of four independent feature-ranking methods. ANOVA F-test evaluates the statistical difference in feature means between classes, capturing linearly separable features. L1-regularized logistic regression (Lasso) identifies features with sparse and stable linear contributions to class prediction, encouraging interpretability and multicollinearity control. XGBoost feature importance estimates non-linear and hierarchical feature interactions by leveraging gradient-boosted decision trees. MultiSURF (ReBATE framework), a nearest-neighbor-based algorithm sensitive to higher-order and non-linear feature dependencies is especially useful for uncovering complex feature interactions. Each method independently ranks features according to its relevance score. The top-k ranked features from each method are assigned to one vote. Votes are then aggregated across methods, and ties are resolved by computing the sum of normalized importance scores (e.g., F-values, absolute logistic coefficients, XGBoost importances). The features with the highest total votes (and tie-sum scores) are retained as the final selected subset. The number of retained features (vfs_k) and the regularization strength of the logistic model (vfs_lr_c) are both optimized within the inner cross-validation. In this study, k ranged from 25 to 100, and vfs_lr_c followed a log-uniform distribution from 10^−3^ to 10^3^. The integration of these four heterogeneous selection mechanisms allows the VFS to capture both linear and non-linear discriminative patterns, improving feature robustness across folds.

A nested cross-validation (CV) approach was implemented to ensure reliable model evaluation. The inner loop optimized model hyperparameters and executed voting feature selection (VFS), while the outer loop assessed model generalization performance.

The outer loop consisted of a 10-fold stratified cross-validation repeated three times. Each outer iteration provided a distinct held-out test subset, unseen during model optimization, used exclusively for performance evaluation. Within each outer training split, an inner cross-validation (*StratifiedKFold*, *n* = 4) was used to perform hyperparameter optimization via Randomized Search (*RandomizedSearchCV*, 20 iterations). The search simultaneously optimized the number of selected features (vfs_k), the L1-regularization strength (vfs_lr_c) in the logistic model within VFS, and the hyperparameters of the XGBoost classifier (e.g., tree depth, number of estimators, learning rate, subsample, and regularization coefficients).

Each configuration was evaluated using multi-metric scoring, including ROC-AUC, accuracy, sensitivity (true positive rate), and specificity (true negative rate).

The AUC was designated as the *refit metric*, meaning that the model achieving the highest inner loop AUC was refitted on the entire outer training data before final evaluation on the outer test set. This hierarchical design ensured that feature selection and model tuning were performed independently within each outer training subset. Moreover, the outer test data remained completely unseen throughout optimization and reported metrics reflect true generalization capability across subjects.

### 2.5. Explainability and Stability Analysis with SHAP

Across all outer folds (10 × 3 = 30 iterations), the best-performing pipeline of each iteration was stored for subsequent explainability analyses using SHAP. To characterize model explainability and the robustness of feature importance across outer folds, we carried out a two-step SHAP-based stability analysis. (i) Global SHAP aggregation and 95% cumulative set. For each outer fold, SHAP values were computed on the held-out test set using the best pipeline refitted on the corresponding training split. We then formed a global summary by averaging mean absolute SHAP values per feature across folds, obtaining a vector *s* sorted in descending order of relevance. The cumulative contribution of the top-*k* features is:

$$C(k)=\frac{\Sigma_{j=1}^{k}{\bar{s}}_{j}}{S}$$

We defined the top-95% SHAP set as the smallest prefix k such that C(k) ≥ 0.95. This yields a compact subset of features that explains ≥95% of the total global importance. (ii) Cross-fold stability via selection frequency.

$${stab}_{j}=100\times \frac{f_{j}}{N}\%$$

Model interpretability was achieved using SHapley Additive exPlanations (SHAP).

SHAP values quantified the contribution of each selected feature to the model’s decision, enabling transparent interpretation of both global feature importance and individual subject predictions. This procedure balances explanatory power (global SHAP mass) with reproducibility (cross-fold selection frequency), yielding a parsimonious subset of features that is interpretable, stable, and generalizable. The relatively large number of features contributing to the cumulative SHAP mass reflects the high dimensionality and partial redundancy of correlated kinematic and graph-theoretical measures, rather than noise or subject-specific effects. The stability-based filtering step was explicitly designed to isolate robust and generalizable features from this distributed contribution.

**Figure 1:**
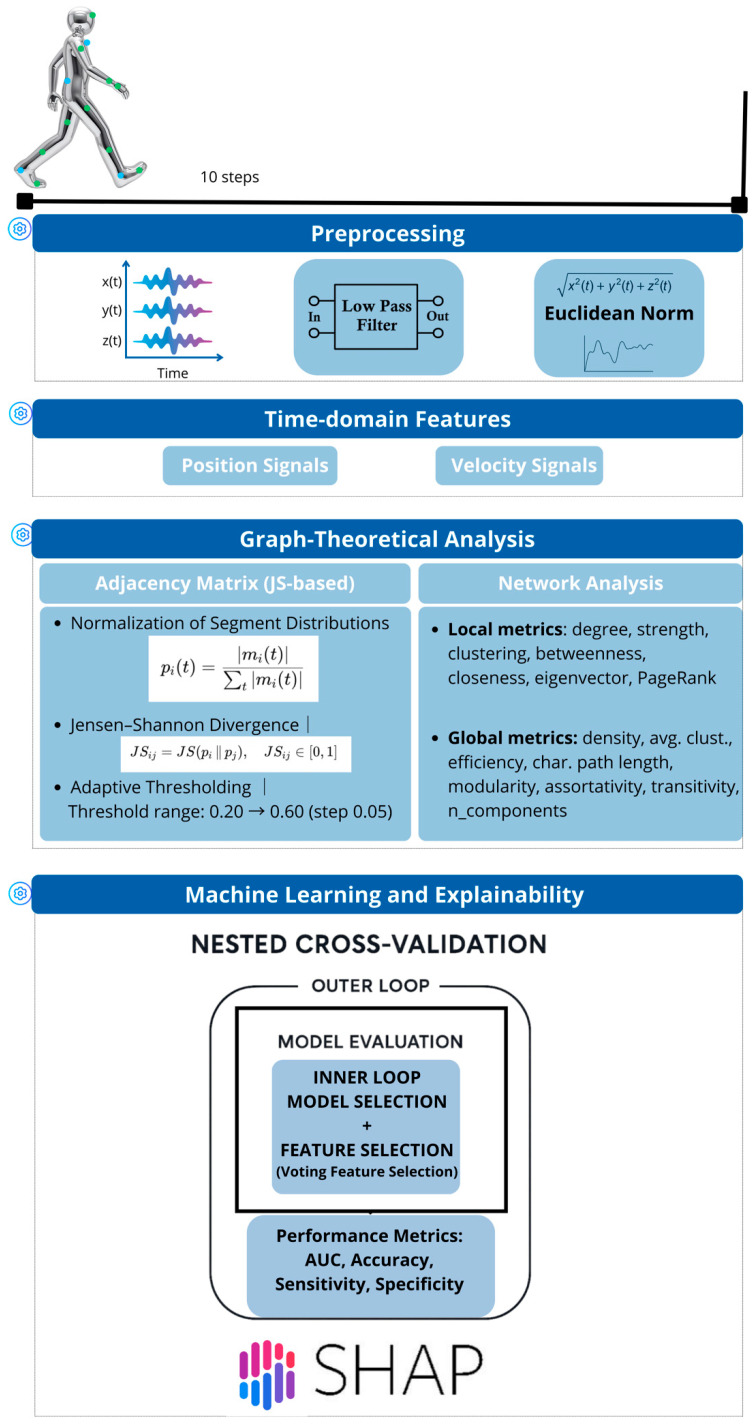
Overview of the inertial AI pipeline for feature extraction, selection, and classification.

The pipeline is summarized in Figure 1.

### 2.6. Statistical Analysis

All statistical analyses were performed using Python 3.10 (SciPy 1.12). Normality of continuous variables was assessed using the Shapiro–Wilk test. Accordingly, group comparisons between patients with PD and HC were performed using Fisher’s exact test for categorical variables (gender distribution) and the Kruskal–Wallis rank-sum test for continuous variables.

Sensitivity power analyses were conducted for the inferential statistics reported in Table 1. For categorical variables, effect size was estimated using Cohen’s *w*, while for continuous variables an approximation based on Cohen’s *d* was adopted, consistent with non-parametric group comparisons. Power was evaluated assuming a two-sided significance level of α = 0.05.

To assess the relationship between model-derived biomechanical features and clinical measures, Spearman’s rank correlation coefficients (ρ) were computed between the SHAP-derived features (selected as the most explanatory and stable across folds) and key clinical scores, including the MDS-UPDRS TOTAL and part III, Hoehn–Yahr (H–Y) stage, disease duration, age at onset, and side-specific motor subscores (bradykinesia, rigidity, and tremor). Correlations with |ρ| ≥ 0.30 and *p* < 0.05 were interpreted as clinically relevant associations.

Classification performance was evaluated using receiver operating characteristic (ROC) analysis within a nested cross-validation framework. The empirical AUC was evaluated relative to chance performance against chance performance (AUC = 0.5). Confidence intervals were estimated across outer cross-validation folds, providing an empirical measure of performance stability and robustness.

## 3. Results

### 3.1. Demographic and Clinical Features

In our study, we enrolled 51 patients with Parkinson’s disease (PD) and 53 healthy controls (HC). The demographic and clinical data are reported in Table 1. Patients with PD were significantly older than HC (*p* < 0.001). They showed a different gender distribution, with a male predominance in PD and a female predominance in HC (*p* < 0.001, Fisher’s exact test). To minimize the potential confounding effects of age and sex, all subsequent analyses were corrected for these variables before statistical testing. Sensitivity power analysis indicated that the available sample size provided adequate statistical power for the group comparisons reported in Table 1. Specifically, the difference in sex distribution between PD and HC groups corresponded to a large effect size (Cohen’s *w* ≈ 0.45), yielding power > 0.95. Likewise, the between-group difference in age at examination showed a very large effect size (Cohen’s *d* ≈ 1.36), associated with power > 0.99.

The disease onset, disease duration, Movement Disorder Society-Unified Parkinson’s Disease Rating Scale (MDS-UPDRS) Total, MDS-UPDRS-III and Hoehn and Yahr (H-Y) showed the typical values associated with the pathology. Side-specific subscores revealed slightly higher bradykinesia in the right compared to the left body side (3.08 ± 3.22 vs. 2.91 ± 3.39), with comparable rigidity and tremor subscores bilaterally. Using a linear univariate approach, the results of the *t*-tests comparing features derived from position and velocity signals (extracted from the motion trackers, MTs) between PD and HC are presented in Supplementary Table S1.

All *p*-values were corrected for multiple comparisons using the false discovery rate (FDR) method. After correction, in the position domain, PD patients exhibited significantly higher mean and standard deviation values than HC, indicating increased signal variability. All features in this domain remained highly significant after FDR correction (*p* < 0.005). In the velocity domain, most features also showed significant group differences (*p* < 0.01). However, a few segments, such as the left upper arm, left upper leg, pelvis, right forearm, right upper leg, and T12, demonstrated moderate significance (*p* < 0.05 *), whereas L5, left shoulder, neck, right upper arm, and T8 did not show any significant differences (*p* > 0.05 *). Overall, PD patients exhibited a lower velocity amplitude compared to HC, reflecting slower and less variable kinematic patterns.

### 3.2. Machine Learning Models and Explainability

**Figure 2:**
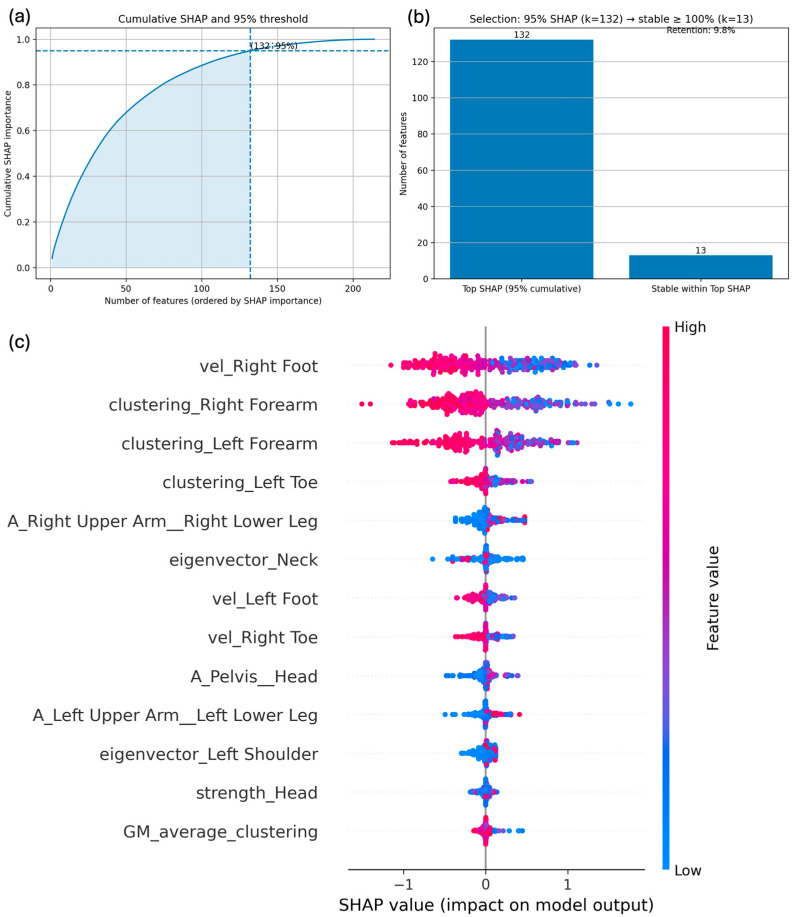
Cumulative SHAP importance curve with 95% threshold (**a**) and selection of stable features (**b**). Beeswarm plot showing the distribution of SHAP values across subjects (**c**).

The results of the machine learning analysis are summarized in Figure 2 and Supplementary Figure S1. Using the XGBoost classifier within the nested cross-validation framework, the model achieved robust performance in distinguishing patients with Parkinson’s disease (PD) from healthy controls (HC). The mean area under the ROC curve (AUC) across outer folds was 0.87 (±0.14), with an accuracy of 0.81 (±0.14), sensitivity of 0.79 (±0.18), and specificity of 0.85 (±0.19) (Supplementary Figure S1). These results demonstrate a stable and well-generalizing classification performance across cross-validation folds. Importantly, the confidence interval of the observed AUC did not include the chance level (AUC = 0.5), indicating statistically significant discrimination between PD and HC. Given the available sample size (51 PD, 53 HC), these considerations support that the study was adequately powered to detect an AUC of this magnitude.

Following feature selection, SHAP analysis was performed to interpret the model decisions and identify the most relevant predictors. The global SHAP summary (Figure 2a) illustrates the cumulative contribution of features to the total model importance. A vertical dashed line marks the 95% cumulative importance threshold, corresponding to 132 features. This gradual accumulation of SHAP importance indicates a distributed contribution across partially redundant features, capturing complementary aspects of movement dynamics and network organization. The subsequent reduction to 13 stable features therefore highlights a parsimonious and reproducible core of predictors, rather than an arbitrary feature selection.

In comparison, the right panel (Figure 2b) summarizes the reduction from these Top-95% SHAP features to the subset of stable features (*n* = 13) that were consistently selected across ≥95% of the folds. The lower panel (Figure 2c) displays a beeswarm plot of the 13 top-ranked features, with positive SHAP values indicating a stronger association with PD and negative values indicating a stronger association with HC. Velocity-related features (e.g., vel_Right Foot, vel_Right Toe, vel_Left Foot) showed lower SHAP values in PD, reflecting reduced movement amplitude and dynamic variability. Similarly, decreases were observed in some local network metrics (e.g., clustering_Left Forearm, clustering_Left Toe) and in the global average clustering metric, suggesting diminished inter-segment coordination in PD. Conversely, features derived from the adjacency matrix (e.g., A_Pelvis_Head, A_Left Upper Arm–Left Lower Leg) and centrality measures (Eigenvector_Neck, Eigenvector_Left Shoulder, strength_Head) exhibited higher SHAP values in PD, indicating stronger or more centralized intersegmental coupling patterns.

**Figure 3:**
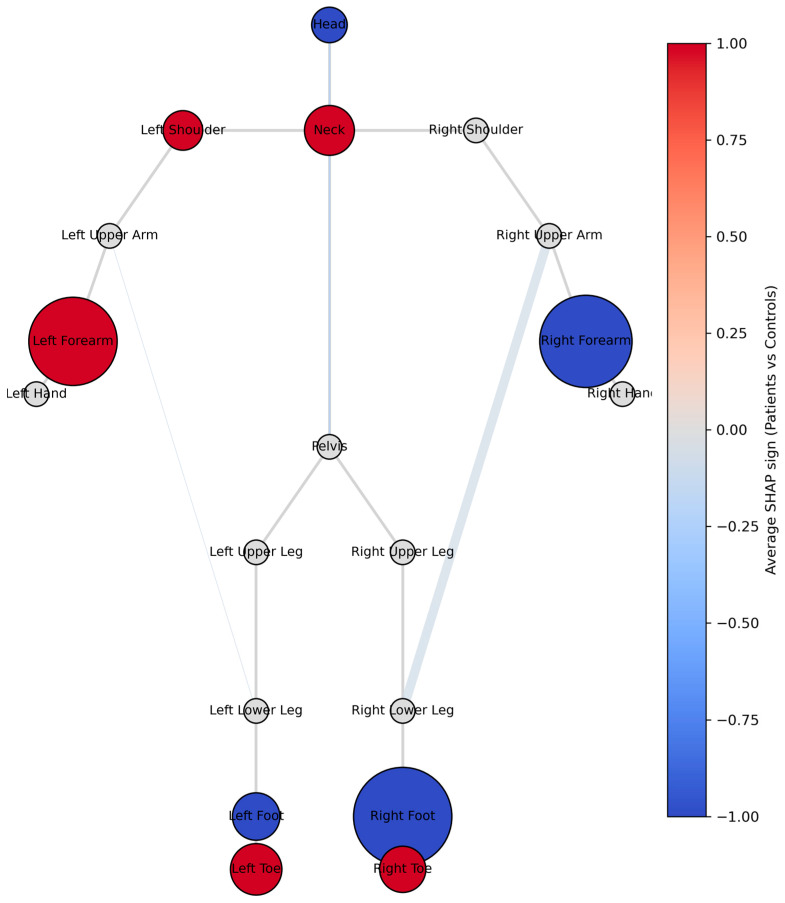
Body network visualization of significant features projected onto a stickman skeleton. Node size represents overall SHAP importance, node color encodes direction of effect (red = stronger association with PD, blue = stronger association with HC), and edge thickness indicates relative connection importance.

In Figure 3, SHAP-derived features are projected onto a body network visualization aligned with a stickman skeleton. Here, node size represents overall SHAP importance, node color encodes the direction of effect (red = stronger association with PD, blue = stronger association with HC), and edge thickness indicates relative connection importance. This visualization reveals that the left forearm and neck contribute most to PD classification, while the right forearm and head are more predictive of HC. Moreover, bilateral Toe segments were strongly associated with PD, whereas bilateral feet characterized HC. Importantly, the connection between upper and lower body segments—particularly between arms and legs—was markedly weakened in PD, suggesting a loss of coordinated visuo-motor integration.

Overall, the combination of classification and explainability analyses indicates that PD is characterized by enhanced rigidity and coupling within central and upper-body segments, together with reduced velocity and coordination in distal limbs. These results support the hypothesis that disrupted intersegmental dynamics and reduced movement adaptability underpin the altered gait and postural control observed in PD.

### 3.3. Correlation

Finally, Supplementary Table S2 presented only the significant (*p*_value < 0.05) Spearman correlations between the 13 best features extracted from SHAP analysis and clinical data, including H-Y, MDS-UDPRS TOTAL, disease duration, and disease onset. Velocity features such as bilateral foot and right toe demonstrated a negative correlation with MDS-UPDRS TOTAL; moreover, right foot and right toe also showed a negative correlation with H-Y and disease onset. Local metrics such as Eigenvector Left Shoulder showed a positive correlation with MDS-UPDRS TOTAL and H-Y, eigenvector neck presented a positive correlation with MDS-UPDRS TOTAL, H-Y and disease duration, although strength head showed a positive correlation only with MDS-UPDRS TOTAL. The two features of the adjacency matrix (left upper arm–left lower leg and pelvis–head) showed a positive correlation only with MDS-UPDRS TOTAL.

Spearman correlation analyses (see Supplementary Table S2) revealed several significant associations between model-derived biomechanical features and clinical measures. Among the global clinical indices, the MDS-UPDRS TOTAL score showed positive correlations with: A_Pelvis_Head (ρ = 0.43, *p* = 0.003), A_Left Upper Arm–Left Lower Leg (ρ = 0.40, *p* = 0.006), Eigenvector Left Shoulder (ρ = 0.36, *p* = 0.012), Eigenvector Neck (ρ = 0.36, *p* = 0.014), and Strength Head (ρ = 0.40, *p* = 0.006). Conversely, negative correlations were observed with velocity-based features of the lower limbs: Vel Right Foot (ρ = −0.40, *p* = 0.006), and Vel Right Toe (ρ = −0.40, *p* = 0.006). The H–Y stage was associated with increased Eigenvector Left Shoulder (ρ = 0.31, *p* = 0.040) and Eigenvector Neck (ρ = 0.32, *p* = 0.035), suggesting that greater global motor severity is associated with greater centrality in upper-body network nodes. Disease duration correlated positively with Strength Head (ρ = 0.30, *p* = 0.040), while earlier disease onset was linked to reduced velocity features of the right foot and toe (ρ = −0.29, *p* = 0.045 for both).

Among side-specific subscores, Bradykinesia Right correlated positively with A_Left Upper Arm–Left Lower Leg (ρ = 0.31, *p* = 0.037) and A_Pelvis_Head (ρ = 0.28, *p* = 0.050), indicating that increased inter-segmental coupling accompanies greater bradykinetic severity. Similarly, Total Right motor scores correlated with Eigenvector Left Shoulder (ρ = 0.29, *p* = 0.050), and Total Left scores were negatively related to Vel Right Foot and Vel Right Toe (ρ = −0.33, *p* = 0.022 for both). Finally, Tremor Total Left correlated inversely with the same velocity-based features (Vel Right Foot: ρ = −0.39, *p* = 0.006; Vel Right Toe: ρ = −0.42, *p* = 0.003), suggesting that reduced distal limb velocity variability corresponds to greater tremor severity.

## 4. Discussion

### 4.1. Main Findings

This study combined whole body kinematic networks with machine learning explainability to identify movement-based biomarkers that discriminate Parkinson’s disease (PD) from healthy controls (HC). The proposed framework demonstrated strong classification performance, with an AUC of 0.87 and an accuracy of 0.81. Additionally, it identified a strict subset of stable and biologically interpretable features. PD is characterized by increased positional variability, indicative of postural instability, along with reduced limb velocity and altered inter-segmental connectivity, reflecting impaired adaptive motor coordination.

To formally quantify these alterations, we employed a graph analysis, which computes metrics such as node degree, clustering, modularity, and efficiency that explained coordination, synchrony, and communication among body segments. This network-based perspective aligns with the growing interest in objective biomarkers of motor symptoms in PD patients. Rather than analyzing movement variables independently, it assesses how the entire movement system is organized, revealing degradation or compensatory mechanisms even when conventional kinematic parameters appear normal.

Consistent with this framework, statistical group comparisons demonstrated greater dispersion in positional signals and reduced velocity magnitudes in PD patients, consistent with bradykinesia and rigidity [2]. Similar kinematic results characterized by reduced speed, amplitude, and segmental coordination, have been reported in gait and upper-limb analyses using inertial sensors [9,29]. The increased variability in position may indicate compensatory oscillations linked to compromised postural control or sensorimotor feedback mechanisms [30]. These findings are in line with prior observations that patients with PD exhibit increased signal irregularity and diminished movement smoothness, both of which tend to worsen as the disease progresses [31]. Through the modeling of inter-segment similarities using Jensen–Shannon divergence, we constructed subject-specific kinematic networks that capture functional coupling across body regions. This approach enables the comparison of signals with potentially variable timing while remaining robust to outliers and noise [32]. Pearson correlation requires temporal synchronization and primarily captures linear relationships, whereas dynamic time warping, although effective for temporal alignment, is computationally expensive and less suitable for fully connected, subject-specific graphs [33]. Mutual information can capture nonlinear dependencies but is more sensitive to noise and probability density estimation, particularly in short or noisy biological time series [34]. In contrast, Jensen–Shannon divergence is symmetric, bounded, and provides an interpretable weighting for constructing robust movement networks that capture inter-segment coordination across subjects [32].

PD networks exhibited increased centrality in proximal nodes, such as the neck, head, and shoulder, while demonstrating decreased efficiency in distal limbs. This pattern indicates reorganization of motor control, wherein the trunk compensates for decreased distal coordination, aligning with alterations in postural–gait coupling and axial rigidity [35,36]. Similar working patterns have been observed in functional networks of PD based on neuroimaging, where overconnectivity of central hubs accompanies peripheral hypoactivity [37].

### 4.2. Clinical Implications

The explainability analysis conducted with SHAP has confirmed that a select subset of thirteen stable features—primarily associated with velocity, clustering, and network centrality—predominantly influenced the classification performance. Lower SHAP values observed in velocity features of the feet and toes indicated slower movement dynamics, while higher SHAP importance attributed to central nodes (neck, head, left forearm) suggested the presence of compensatory rigidity and coupling. These findings corroborate previous biomechanical research, which demonstrate that reduced distal mobility alongside compensatory proximal stiffness are characteristic signatures of PD motor dysfunction [38,39,40].

The body network visualization further demonstrated an inter-limb asymmetry—left forearm dominance for PD versus right forearm contribution for HC—suggesting possible lateralized control deterioration, in agreement with reports of asymmetric motor onset and coordination deficits [41]. The loss of strong upper–lower body connectivity seen in PD likely reflects a breakdown of visuo-motor integration and interlimb synchronization, mechanisms mediated by subcortical–cerebellar loops [42].

Correlations between SHAP features and clinical scores reinforced their physiological interpretability. Features reflecting higher network strength and eigenvector centrality correlated positively with the MDS-UPDRS total score and H–Y stage, indicating that more centralized and rigid coordination patterns are associated with greater disease severity. Conversely, decreased velocity features correlated with bradykinesia and tremor subscores, supporting the view that velocity variability may serve as a sensitive marker of dopaminergic motor impairment [43,44]. These associations suggest that explainable ML features can connect quantitative movement biomarkers with clinical motor scales, potentially improving patient monitoring and therapeutic assessment.

### 4.3. Strengths and Limitations

The nested cross-validation and multi-criteria feature voting framework were used to improve model robustness and reduce overfitting, a common challenge in biomedical ML [45]. The use of explainable AI may also aid individual-level interpretation of motor impairment and guide personalized rehabilitation interventions. However, these strengths must be interpreted in light of certain limitations. First, as this is a pilot study, larger and more demographically balanced cohorts are required to confirm generalizability and to further mitigate potential age- and sex-related confounding effects. Given the higher age and relatively long disease duration of the PD cohort, the present results primarily reflect established Parkinsonian motor dysfunction rather than early diagnostic markers, despite age- and sex-correction.

Second, the study focused on a specific set of motor tasks, kinematic networks derived from other tasks or sensor configurations may capture complementary aspects of motor dysfunction. Third, although SHAP-based explainability improves interpretability, it remains dependent on the underlying model and feature space. Finally, frequency-domain features, which may capture tremor-related oscillatory activity, were not included in the present analysis; future studies could investigate their complementary value for phenotype-specific characterization.

### 4.4. Future Research

Future studies should address these limitations by conducting larger, multi-center, longitudinal investigations and by integrating multimodal data, including neuroimaging and early-stage cohorts. Moreover, future research should investigate task-specific networks, incorporate nonlinear dynamical metrics, integrate neuroimaging-derived connectivity to reveal kinematic and neural alterations, and include frequency-domain features for phenotype-specific characterization.

## 5. Conclusions

In conclusion, this study shows that whole body kinematic networks combined with explainable machine learning can discriminate PD from healthy controls with good accuracy, while retaining physiological interpretability. Increased positional variability, reduced distal limb velocity, and reorganization of inter-segment connectivity emerged as biomarkers based on movement, in line with postural instability, bradykinesia, and compensatory proximal rigidity. The association between these features and clinical scores suggests that kinematic network biomarkers may provide an objective, quantitative complement to standard motor assessment, with potential applications in monitoring disease severity and tailoring rehabilitation in PD.

## Figures and Tables

**Table 1:** Demographic and clinical data of patients with Parkinson’s disease and control subjects.

Data	PD (N = 51)	HC (N = 53)	*p*-Value
Gender, (M/F)	37/14	19/34	<0.001 ^a^
Age at examination, ys ^b^	69.6 ± 8.67	51.3 ± 17.03	<0.001 ^c^
Disease onset ^b^	62.0 ± 8.96	-	-
Disease duration, ys ^b^	7.6 ± 4.49	-	-
MDS UPDRS TOTAL ^b^	29.9 ± 19.0	-	-
MDS UPDRS-III ^b^	17.0 ± 11.0	-	-
H-Y score ^b^	1.54 ± 0.60	-	-
Bradykinesia_tot_left	2.91 ± 3.39	-	-
Bradykinesia_tot_right	3.08 ± 3.22	-	-
Rigidity_tot_left	0.94 ± 0.94	-	-
Rigidity_tot_right	1.13 ± 1.19	-	-
Tremor_tot_left	0.61 ± 1.37	-	-
Tremor_tot_right	0.74 ± 1.15	-	-
Total_left	4.46 ± 4.80	-	-
Total_right	4.95 ± 4.62	-	-

Abbreviations: PD = Parkinson’s disease; HC = Healthy controls; MDS-UPDRS-III = Movement Disorder Society-Unified Parkinson’s Disease Rating Scale-part III (Motor Examination); H-Y = Hoehn and Yahr; ^a^ Fisher’s exact test. ^b^ Data are expressed as mean ± standard deviation. ^c^ Kruskal–Wallis’s rank test.

## Data Availability

The data that support the results of this study are available from the corresponding author upon reasonable request.

## Supplementary Materials

The following supporting information can be downloaded at: https://www.mdpi.com/article/10.3390/bioengineering13020151/s1, Figure S1: Receiver operating characteristic (ROC) curve showing classification performance (AUC) across outer cross-validation folds; Table S1: Data of position and velocity of the sensors in PD and HC; Table S2: Spearman correlations between SHAP-derived features and clinical variables.

## References

[1] van Rooden S.M., Visser M., Verbaan D., Marinus J., van Hilten J.J. (2009). Motor patterns in Parkinson’s disease: A data-driven approach. Mov. Disord..

[2] Bologna M., Espay A.J., Fasano A., Paparella G., Hallett M., Berardelli A. (2023). Redefining Bradykinesia. Mov. Disord..

[3] Kehnemouyi Y.M., Coleman T.P., Tass P.A. (2024). Emerging wearable technologies for multisystem monitoring and treatment of Parkinson’s disease: A narrative review. Front. Netw. Physiol..

[4] Brognara L., Mazzotti A., Zielli S.O., Arceri A., Artioli E., Traina F., Faldini C. (2024). Wearable Technology Applications and Methods to Assess Clinical Outcomes in Foot and Ankle Disorders: Achievements and Perspectives. Sensors.

[5] Caballol N., Bayés À., Prats A., Martín-Baranera M., Quispe P. (2023). Feasibility of a wearable inertial sensor to assess motor complications and treatment in Parkinson’s disease. PLoS ONE.

[6] Calomino C., Gramigna V., Bianco M.G., Cristofaro A., Quattrone A., Quattrone A. A Quantitative Kinematic Evaluation of Postural Response in Parkinson’s Disease Subtypes. Proceedings of the 2023 International Workshop on Biomedical Applications, Technologies and Sensors (BATS).

[7] Dietz V., Zijlstra W., Prokop T., Berger W. (1995). Leg muscle activation during gait in Parkinson’s disease: Adaptation and interlimb coordination. Electroencephalogr. Clin. Neurophysiol./Electromyogr. Mot. Control.

[8] Allen N.E., Canning C.G., Sherrington C., Fung V.S.C. (2009). Bradykinesia, muscle weakness and reduced muscle power in Parkinson’s disease. Mov. Disord..

[9] Rovini E., Maremmani C., Cavallo F. (2017). How Wearable Sensors Can Support Parkinson’s Disease Diagnosis and Treatment: A Systematic Review. Front. Neurosci..

[10] Bassett D.S., Sporns O. (2017). Network neuroscience. Nat. Neurosci..

[11] Lodin J., Jelínek M., Sameš M., Vachata P. (2022). Quantitative Gait Analysis of Patients with Severe Symptomatic Spinal Stenosis Utilizing the Gait Profile Score: An Observational Clinical Study. Sensors.

[12] Calomino C., Quattrone A., Bianco M.G., Nisticò R., Buonocore J., Crasà M., Vaccaro M.G., Sarica A., Quattrone A. (2024). Combined cortical thickness and blink reflex recovery cycle to differentiate essential tremor with and without resting tremor. Front. Neurol..

[13] Bianco M.G., Caligiuri M.E., Calomino C., Bonacci M.C., Aquila V., Buonocore J., Augimeri A., Sarica A., Vaccaro M.G., Quattrone A. (2025). Volumetric Assessment and Graph Theoretical Analysis of Thalamic Nuclei in Essential Tremor. Brain Behav..

[14] Zhang Y., Nie L. (2024). Human motion similarity evaluation based on deep metric learning. Sci. Rep..

[15] Ali L., Leung M.-F., Khan M.A., Nour R., Imrana Y., Vasilakos A.V. (2025). ChiGa-Net: A genetically optimized neural network with refined deeply extracted features using statistical score for trustworthy Parkinson’s disease detection. Neurocomputing.

[16] He J., Zhuang L., Chaurasia A., Najafi A. (2024). Investigating the Application of Stress Wave Factors in Machine Learning for Delamination Location Prediction in a Composite Laminate. AIAA SCITECH 2024 Forum.

[17] Mahajan H., Banerjee S. (2022). A machine learning framework for guided wave-based damage detection of rail head using surface-bonded piezo-electric wafer transducers. Mach. Learn. Appl..

[18] Kovar L., Gleicher M., Pighin F. (2023). Motion Graphs. Seminal Graphics Papers: Pushing the Boundaries.

[19] Jalata I.K., Truong T.-D., Allen J.L., Seo H.-S., Luu K. (2021). Movement Analysis for Neurological and Musculoskeletal Disorders Using Graph Convolutional Neural Network. Future Internet.

[20] Naro A., Calabrò R.S., La Rosa G., Andronaco V.A., Billeri L., Lauria P., Bramanti A., Bramanti P. (2019). Toward understanding the neurophysiological basis of peripersonal space: An EEG study on healthy individuals. PLoS ONE.

[21] Rashnu A., Salimi-Badr A., Rashnu A., Salimi-Badr A. (2024). Integrative Deep Learning Framework for Parkinson’s Disease Early Detection using Gait Cycle Data Measured by Wearable Sensors: A CNN-GRU-GNN Approach. arXiv.

[22] Postuma R.B., Berg D., Stern M., Poewe W., Olanow C.W., Oertel W., Obeso J., Marek K., Litvan I., Lang A.E. (2015). MDS clinical diagnostic criteria for Parkinson’s disease. Mov. Disord..

[23] Goetz C.G., Tilley B.C., Shaftman S.R., Stebbins G.T., Fahn S., Martinez-Martin P., Poewe W., Sampaio C., Stern M.B., Dodel R. (2008). Movement Disorder Society-sponsored revision of the Unified Parkinson’s Disease Rating Scale (MDS-UPDRS): Scale presentation and clinimetric testing results. Mov. Disord..

[24] Movella (Xsens Technologies) (2020). Xsens MVN Animate. https://www.movella.com/motion-capture/xsens-mvn-animate.

[25] Li P., Yan H., Lu X. (2023). A Siamese neural network for learning the similarity metrics of linear features. Int. J. Geogr. Inf. Sci..

[26] Duong T. (2007). ks: Kernel Density Estimation and Kernel Discriminant Analysis for Multivariate Data in R. J. Stat. Softw..

[27] Fornito A., Zalesky A., Breakspear M. (2015). The connectomics of brain disorders. Nat. Rev. Neurosci..

[28] Fornito A., Zalesky A., Bullmore E.T. (2016). Fundamentals of Brain Network Analysis.

[29] Del Din S., Godfrey A., Mazzà C., Lord S., Rochester L. (2016). Free-living monitoring of Parkinson’s disease: Lessons from the field. Mov. Disord..

[30] Herman T., Weiss A., Brozgol M., Giladi N., Hausdorff J.M. (2014). Gait and balance in Parkinson’s disease subtypes: Objective measures and classification considerations. J. Neurol..

[31] Patel S., Lorincz K., Hughes R., Huggins N., Growdon J., Standaert D., Akay M., Dy J., Welsh M., Bonato P. (2009). Monitoring Motor Fluctuations in Patients with Parkinson’s Disease Using Wearable Sensors. IEEE Trans. Inf. Technol. Biomed..

[32] Gong L., Wang J. Complexity analysis of electroencephalogram signal based on Jensen-Shannon divergence. Proceedings of the 2013 6th International Conference on Biomedical Engineering and Informatics.

[33] Shih P.J., Saadat H., Parameswaran S., Gamaarachchi H. (2022). Efficient real-time selective genome sequencing on resource-constrained devices. Gigascience.

[34] Faes L., Porta A., Nollo G., Javorka M. (2016). Information Decomposition in Multivariate Systems: Definitions, Implementation and Application to Cardiovascular Networks. Entropy.

[35] Plotnik M., Giladi N., Hausdorff J.M. (2008). Bilateral coordination of walking and freezing of gait in Parkinson’s disease. Eur. J. Neurosci..

[36] Mirelman A., Bonato P., Camicioli R., Ellis T.D., Giladi N., Hamilton J.L., Hass C.J., Hausdorff J.M., Pelosin E., Almeida Q.J. (2019). Gait impairments in Parkinson’s disease. Lancet Neurol..

[37] Sreenivasan K., Bonato P., Camicioli R., Ellis T.D., Giladi N., Hamilton J.L., Hass C.J., Hausdorff J.M., Pelosin E., Almeida Q.J. (2023). Topological reorganization of functional hubs in patients with Parkinson’s disease with freezing of gait. J. Neuroimaging.

[38] Suzuki M., Mitoma H., Yoneyama M. (2017). Quantitative Analysis of Motor Status in Parkinson’s Disease Using Wearable Devices: From Methodological Considerations to Problems in Clinical Applications. Park. Dis..

[39] Verschueren S.M.P., Swinnen S.P., Dom R., De Weerdt W. (1997). Interlimb coordination in patients with Parkinson’s disease: Motor learning deficits and the importance of augmented information feedback. ExBrain Res..

[40] Trabassi D., Serrao M., Varrecchia T., Ranavolo A., Coppola G., De Icco R., Tassorelli C., Castiglia S.F. (2022). Machine Learning Approach to Support the Detection of Parkinson’s Disease in IMU-Based Gait Analysis. Sensors.

[41] Cubo E., Martín P.M., Martin-Gonzalez J.A., Rodríguez-Blázquez C., Kulisevsky J. (2010). Motor laterality asymmetry and nonmotor symptoms in Parkinson’s disease. Mov. Disord..

[42] Wu T., Hallett M. (2013). The cerebellum in Parkinson’s disease. Brain.

[43] Park H., Shin S., Youm C., Cheon S.-M. (2024). Deep learning-based detection of affected body parts in Parkinson’s disease and freezing of gait using time-series imaging. Sci. Rep..

[44] Salarian A., Russmann H., Wider C., Burkhard P.R., Vingerhoets F.J.G., Aminian K. (2007). Quantification of Tremor and Bradykinesia in Parkinson’s Disease Using a Novel Ambulatory Monitoring System. IEEE Trans. Biomed. Eng..

[45] Chicco D., Jurman G. (2020). The advantages of the Matthews correlation coefficient (MCC) over F1 score and accuracy in binary classification evaluation. BMC Genom..

