# Supplementary material for: Similarity Gait Networks with XAI for Parkinson’s Disease Classification: A Pilot Study
Source: Bioengineering (Basel). 2026 Jan 28;13(2):151. doi: 10.3390/bioengineering13020151 (PMC12937932; doi:10.3390/bioengineering13020151)
Supplement: Supplementary file 1 [file bioengineering-13-00151-s001.zip › bioengineering-4083414-supplementary.pdf]

Supplementary Materials

Supplementary Figure S1. Receiver Operating Characteristic (ROC) curve showing classification performance (AUC) across outer cross-validation folds.

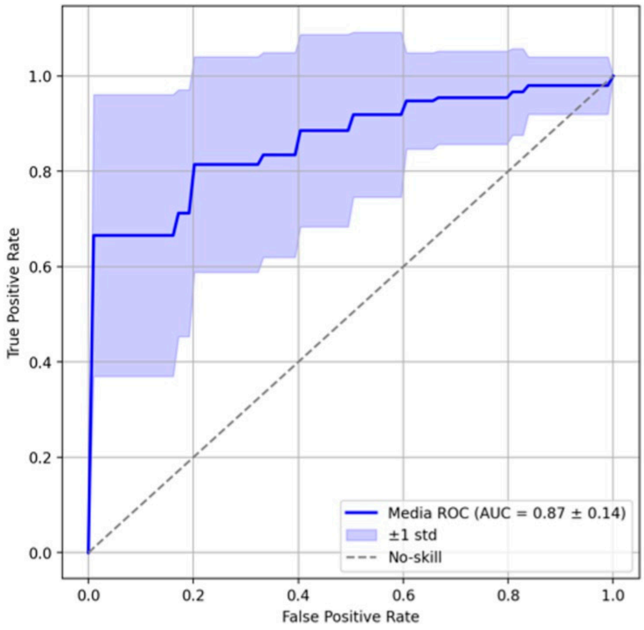

Metrics

ROC AUC	0.87 (0.14)
Accuracy	0.81 (0.14)
Sensitivity	0.79 (0.18)
Specificity	0.85 (0.19)

Supplementary Table S1. Data of position and velocity of the sensors in PD and HC.

Features	PD	HC	p_value	p_FDR
**pos_Head[a]**	1.09 ± 0.346	0.904 ± 0.217	0.001[b]	0.0021[b]
**pos_L3[a]**	1.14 ± 0.372	0.934 ± 0.222	<0.001[b]	0.0021[b]
**pos_L5[a]**	1.15 ± 0.378	0.941 ± 0.222	<0.001[b]	0.0021[b]
**pos_Left Foot[a]**	1.20 ± 0.417	0.985 ± 0.217	0.001[b]	0.0021[b]
**pos_Left Forearm[a]**	1.13 ± 0.363	0.939 ± 0.213	0.002[b]	0.0037[b]
**pos_Left Hand[a]**	1.15 ± 0.375	0.953 ± 0.212	0.001[b]	0.0021[b]
**pos_Left Lower Leg[a]**	1.18 ± 0.395	0.963 ± 0.221	<0.001[b]	0.0021[b]
**pos_Left Shoulder[a]**	1.11 ± 0.355	0.916 ± 0.217	0.001[b]	0.0021[b]
**pos_Left Toe[a]**	1.20 ± 0.409	0.978 ± 0.234	<0.001[b]	0.0021[b]
**pos_Left Upper Arm[a]**	1.10 ± 0.350	0.923 ± 0.209	0.003[b]	0.0049[b]
**pos_Left Upper Leg[a]**	1.16 ± 0.378	0.950 ± 0.216	<0.001[b]	0.0021[b]
**pos_Neck[a]**	1.10 ± 0.352	0.912 ± 0.217	0.001[b]	0.0021[b]
**pos_Pelvis[a]**	1.16 ± 0.384	0.947 ± 0.224	<0.001[b]	0.0021[b]
**pos_Right Foot[a]**	1.22 ± 0.425	0.987 ± 0.235	<0.001[b]	0.0021[b]
**pos_Right Forearm[a]**	1.16 ± 0.380	0.934 ± 0.218	<0.001[b]	0.0021[b]
**pos_Right Hand[a]**	1.18 ± 0.390	0.954 ± 0.218	<0.001[b]	0.0021[b]
**pos_Right Lower Leg[a]**	1.20 ± 0.407	0.966 ± 0.229	<0.001[b]	0.0021[b]
**pos_Right Shoulder[a]**	1.11 ± 0.358	0.917 ± 0.218	0.001[b]	0.0021[b]
**pos_Right Toe[a]**	1.22 ± 0.411	0.975 ± 0.247	<0.001[b]	0.0021[b]
**pos_Right Upper Arm[a]**	1.13 ± 0.367	0.917 ± 0.217	<0.001[b]	0.0021[b]
**pos_Right Upper Leg[a]**	1.17 ± 0.392	0.947 ± 0.225	<0.001[b]	0.0021[b]
**pos_T8[a]**	1.13 ± 1.05	0.928 ± 0.220	0.001[b]	0.0021[b]
**pos_T12[a]**	1.12 ± 1.04	0.922 ± 0.219	0.001[b]	0.0021[b]
**vel_Head[a]**	0.95 ± 3.11	0.574 ± 0.169	0.381[b]	0.428[b]
**vel_L3[a]**	0.52 ± 0.164	0.578 ± 0.162	0.072[b]	0.093[b]
**vel_L5[a]**	0.521 ± 0.163	0.578 ± 0.162	0.074[b]	<0.095[b]
**vel_Left Foot[a]**	1.213 ± 0.327	1.525 ± 0.276	<0.001[b]	0.00121[b]
**vel_Left Forearm[a]**	0.529 ± 0.159	0.609 ± 0.134	0.006[b]	0.009[b]
**vel_Left Hand[a]**	0.585 ± 0.186	0.710 ± 0.183	<0.001[b]	0.0021[b]
**vel_Left Lower Leg[a]**	0.695 ± 0.188	0.827 ± 0.114	<0.001[b]	0.0021[b]

**vel_Left Shoulder[a]**	0.505 ± 0.174	0.575 ± 0.162	0.037[b]	0.051[b]
**vel_Left Toe[a]**	1.213 ± 0.327	1.525 ± 0.276	<0.001[b]	0.0021[b]
**vel_Left Upper Arm[a]**	0.505 ± 0.165	0.574 ± 0.148	0.025[b]	0.035[b]
**vel_Left Upper Leg [a]**	0.531 ± 0.164	0.605 ± 0.151	0.019[b]	0.028[b]
**vel_Neck[a]**	0.511 ± 0.163	0.571 ± 0.151	0.054[b]	0.071[b]
**vel_Pelvis[a]**	0.515 ± 0.172	0.597 ± 0.160	0.013[b]	0.019[b]
**vel_Right Foot[a]**	1.216 ± 0.336	1.558 ± 0.255	<0.001[b]	0.0021[b]
**vel_Right Forearm[a]**	0.501 ± 0.164	0.582 ± 0.145	0.008[b]	0.013[b]
**vel_Right Hand[a]**	0.566 ± 0.195	0.680 ± 0.185	0.003[b]	0.005[b]
**vel_Right Lower Leg[a]**	0.701 ± 0.190	0.821 ± 0.109	<0.001[b]	<0.001[b]
**vel_Right Shoulder[a]**	0.503 ± 0.171	0.567 ± 0.165	0.055[b]	0.055[b]
**vel_Right Toe[a]**	1.216 ± 0.336	1.558 ± 0.255	<0.001[b]	<0.001[b]
**vel_Right Upper Arm[a]**	0.486 ± 0.172	0.547 ± 0.151	0.058[b]	0.058[b]
**vel_Right Upper Leg[a]**	0.528 ± 0.167	0.590 ± 0.149	0.049[b]	0.049[b]
**vel_T8[a]**	0.517 ± 0.164	0.574 ± 0.159	0.073[b]	0.073[b]
**vel_T12[a]**	0.513 ± 0.174	0.582 ± 0.165	0.041[b]	0.041[b]

[a]Data are expressed as mean ± standard deviation. [b] t-test. PD = Parkinson’s disease; HC = Healthy controls.

Supplementary Table S2. Spearman correlations between SHAP- derived features and clinical variables

	A_Left Upper Arm__Left Lower Leg	A_Pelvis_Head	Eigenvector Left Shoulder	Eigenvector Neck	Strength Head	Vel Left Foot	Vel Right Foot	Vel Right Toe
**H-Y**			0.31 (p=0.040)	0.32 (p=0.035)			-0.32 (p=0.038)	-0.32 (p=0.038)
**Mds Updrs-Total**	0.40 (p=0.006)	0.43 (p=0.003)	0.36 (p=0.012)	0.36 (p=0.014)	0.40 (p=0.006)	-0.34 (p=0.018)	-0.40 (p=0.006)	-0.40 (p=0.006)
**Disease Duration**				0.30 (p=0.040)				
**Disease Onset**							-0.29 (p=0.045)	-0.29 (p=0.045)
**Bradykinesia_ Tot_Right**	0.31 (p=0.037)	0,28 (p=0.05)						
**Bradykinesia_ Tot_Left**							-0.29 (p=0.05)	-0.29 (p=0.05)
**Total_Right**	0.29 (p=0.050)							
**Total_Left**							-0.33 (p=0.022)	-0.33 (p=0.022)
**Tremor_ Tot_Left**						-0.39 (p=0.006)	-0.42 (p=0.003)	-0.42 (p=0.003)

Values represent correlation coefficient (p-value).
